# Orthopedic Manifestations of Syringomyelia: A Comprehensive Review

**DOI:** 10.3390/jcm14093145

**Published:** 2025-05-01

**Authors:** Mohamad Fadila, Geva Sarrabia, Shay Shapira, Eyal Yaacobi, Yuval Baruch, Itzhak Engel, Nissim Ohana

**Affiliations:** 1 Orthopedic Department, Meir Medical Center, Kfar Saba, Affiliated to Faculty of Health Sciences and Medicine, Tel-Aviv University, Tel Aviv 6997801, Israel; mohamad.fadila@gmail.com (M.F.); geva017@gmail.com (G.S.); shay_shapira@yahoo.com (S.S.); yaacobi.eyal@gmail.com (E.Y.); 2 Spine Surgery Unit, Meir Medical Center, Affiliated to Faculty of Health Sciences and Medicine, Tel-Aviv University, Tel Aviv 6997801, Israel; yuvalba@clalit.org.il (Y.B.); itzhak.engel@clalit.org.il (I.E.)

**Keywords:** syringomyelia, spinal cord diseases, Charcot joint, neurogenic arthropathy, scoliosis, orthopedic procedures, cerebrospinal fluid, Chiari malformation, spasticity, neuropathic pain

## Abstract

**Background**: Syringomyelia is a complex neurological disorder characterized by a fluid-filled cavity (syrinx) within the spinal cord, frequently resulting from altered cerebrospinal fluid (CSF) dynamics. While its clinical manifestations are diverse, orthopedic complications such as scoliosis, pes cavus, and Charcot arthropathy may represent early diagnostic clues yet are often under-recognized. **Methods**: This comprehensive review synthesizes the current literature on the pathophysiology, clinical presentation, diagnostic strategies, and management approaches of syringomyelia, with a specific emphasis on its orthopedic manifestations. Additionally, we present a detailed case of neuropathic shoulder arthropathy associated with advanced syringomyelia. **Results**: Orthopedic involvement in syringomyelia includes progressive spinal deformities and neurogenic joint destruction, particularly affecting the shoulder and elbow. Scoliosis is frequently observed, especially in association with Chiari malformations, and may precede neurologic diagnosis. Charcot joints result from impaired proprioception and protective sensation. The case presented illustrates the diagnostic challenges and therapeutic dilemmas in managing advanced neuro-orthopedic complications in syringomyelia. **Conclusions**: Syringomyelia should be considered in the differential diagnosis of atypical musculoskeletal presentations. Early recognition and multidisciplinary management are essential to prevent irreversible orthopedic sequelae. Conservative treatment remains the mainstay in stable cases, while surgery is reserved for progressive disease. Orthopedic assessment plays a pivotal role in the diagnostic pathway and long-term care of affected patients.

## 1. Introduction

**Figure 1:**
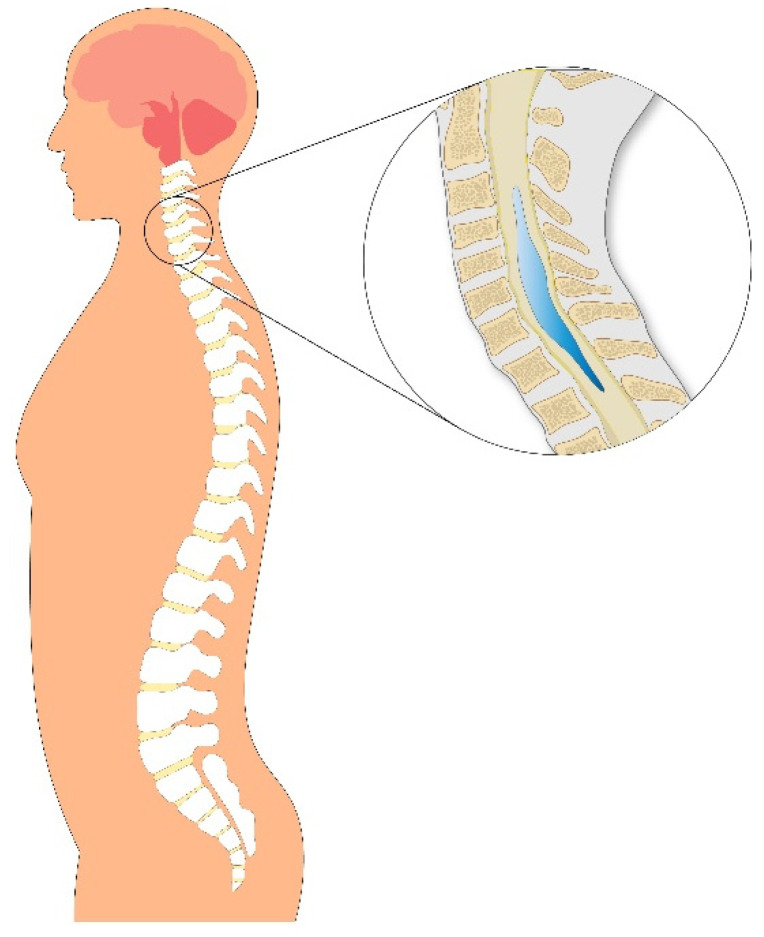
Schematic illustration of syringomyelia demonstrating the formation of a fluid-filled syrinx cavity within the spinal cord. The zoomed-in view highlights the syrinx expanding within the thoracic spinal cord, compressing adjacent neural structures and disrupting cerebrospinal fluid (CSF) dynamics—one of the primary mechanisms underlying the development of syringomyelia.

Syringomyelia is an uncommon neurological disorder characterized by the presence of a fluid-filled cavity, or syrinx, within the spinal cord. This cavity typically contains fluid similar in composition to cerebrospinal fluid (CSF). The terms syrinx and syringomyelia are often used interchangeably in the literature. A closely related entity, hydromyelia, refers to a dilated central canal that persists beyond birth. Other terms such as “dilated central canal”, “hydromyelia”, and “slit-like syrinx” are frequently used to describe similar pathological findings within the spinal cord [1]. Syringomyelia can arise from various etiologies, including infectious adhesive arachnoiditis, spinal cord lesions, and traumatic injury. It is most commonly associated with Chiari malformation type 1 (CM-1). With the increasing use of magnetic resonance imaging (MRI) in the evaluation of back and neck pain, syringomyelia is often discovered incidentally (Figure 1).

Symptomatic cases typically present with sensory disturbances, particularly involving pain and temperature sensation. The natural history of syringomyelia is variable and unpredictable, often characterized by alternating periods of stability and progression [2]. The underlying pathophysiology of syringomyelia is primarily attributed to the obstruction of CSF flow within the spinal subarachnoid space. This obstruction can be broadly categorized into two types [2]:

A. Complete blockage, where the CSF pressure pulse wave is halted at the point of obstruction and transmitted into the spinal cord;
B. Partial blockage, which allows some degree of CSF flow and is explained by two hemodynamic principles.

The Bernoulli principle posits that as CSF flows through a narrowed channel, velocity increases and pressure decreases. Complementing this is the Venturi effect, wherein rapid fluid movement creates a suction force. These mechanisms contribute to parenchymal dilation beneath the obstruction, facilitating CSF accumulation and gradual syrinx formation through the expansion of extracellular spaces. Evidence of a partial obstruction may be inferred through craniospinal MRI findings, particularly when features of communicative hydrocephalus are present [3].

It is also believed that disturbances in CSF dynamics between the cranial and spinal compartments—particularly during the cardiac systolic and diastolic cycles—play a central role in syrinx development. Nonetheless, the primary etiologies of syringomyelia are closely tied to the underlying pathologic processes [4]. Consequently, current classification systems identify five distinct etiological categories based on pathophysiology, four of which are typically associated with syringomyelia [5]:

1. Abnormal CSF flow dynamics associated with hindbrain malformations.

1. Intramedullary or perimedullary congenital or acquired tissue damage.
2. Syringomyelia resulting from intramedullary tumors (primarily ependymomas or hemangioblastomas) with secretory activity.
3. Syringomyelia secondary to supratentorial hydrocephalus.

Understanding the diverse etiologies, pathophysiological mechanisms, and clinical implications of syringomyelia is essential for accurate diagnosis and effective management. Given its potential to cause significant neurological and orthopedic complications, including musculoskeletal manifestations such as Charcot arthropathy, a comprehensive review is warranted. The following sections will explore the various types, classifications, clinical presentations, and radiographic features of syringomyelia, with a particular emphasis on its orthopedic relevance.

## 2. Subtypes and Morphological Variants of Syringomyelia

Syringomyelia encompasses a heterogeneous group of spinal cord cavitations, with various clinical and radiographic subtypes distinguished by their underlying etiology and morphological presentations. Among these, non-communicating syringomyelia is considered the most prevalent. Based on etiological and anatomical distinctions, four principal types have been described [6]:

1. Type I: Associated with hindbrain abnormalities such as Chiari malformation type I (CM-1), basilar invagination, or other lesions obstructing CSF flow at the foramen magnum, leading to central canal dilatation and syrinx formation.

4. Type II: Often considered idiopathic, this type also arises at the foramen magnum but without any identifiable obstruction.
5. Type III: Secondary to intraspinal disorders, such as tumors, traumatic myelopathy, arachnoiditis, pachymeningitis, or myelomalacia, which directly affect the spinal cord parenchyma.
6. Type IV: Referred to as pure hydromyelia, characterized by central canal dilatation without clear disruption to surrounding tissue.

In parallel, various morphological classifications have been proposed to describe the spectrum of spinal cord cavities. These include holocord syringomyelia (extending throughout the cord), localized cysts or spindles, and central canal dilatations, which may be seen even in fully developed nervous systems. Although the terminology remains somewhat inconsistent in the literature, distinctions are often made between the following:

- Hydromyelia, typically referring to a dilated ependymal-lined central canal.
- Syringomyelia, denoting glial-lined cavities that disrupt the cord parenchyma.

Radiologists generally classify larger, eccentric, or irregular cavities as syringomyelia, whereas thin, symmetrical, centrally located dilatations are often labeled as hydromyelia. Despite these distinctions, many experts consider these variations part of a common spectrum, and the term “syringomyelia” remains the most broadly applicable descriptor.

Notably, some debate persists over semantic nuances, including the appropriate plural form—syringes versus syrinxes—although such linguistic distinctions are largely academic and do not affect clinical interpretation [7].

## 3. Prevalence

The reported prevalence of syringomyelia varies across populations. It is estimated to affect approximately 1.94 per 100,000 individuals in Japan and 8.4 per 100,000 individuals in Western countries [2,3]. Although syringomyelia is not uncommon in children—particularly those with congenital anomalies such as Chiari malformation type I (CM-1), tethered cord, or arachnoiditis—it is more frequently observed in young adults. The most common underlying etiology remains aberrant cerebrospinal fluid (CSF) flow across the foramen magnum, accounting for nearly 70% of cases. This is followed by arachnoiditis, and, less frequently, post-traumatic syringomyelia, which accounts for fewer than 4% of cases [1]. Discrepancies in prevalence estimates are likely influenced by differences in study populations and variability in the sensitivity of Chiari malformation detection across imaging protocols. Notably, children and young adults are more likely to exhibit low cerebellar tonsil position and Chiari malformation on MRI compared to older individuals. Studies involving larger cohorts of older patients tend to report lower prevalence rates, highlighting the role of age and imaging interpretation in epidemiologic variation [8].

## 4. Etiology

Syringomyelia is a multifactorial condition with a range of underlying causes that contribute to syrinx formation. These etiologies can be broadly categorized into Chiari-related, primary spinal, and acquired or iatrogenic risk factors. Understanding these distinct mechanisms is essential for guiding diagnostic evaluation and management strategies.

### 4.1. Chiari-Related Syringomyelia

Chiari malformation, particularly Chiari malformation type I (CM-I), is the most common cause of syringomyelia. It is estimated that approximately 69% of adults and 40% of children with CM-I will develop a syrinx [9]. Historically, the term “communicating syringomyelia” was used to describe this subtype, based on early theories suggesting a direct connection between the fourth ventricle and the syrinx cavity. This concept formed the basis of Gardner’s surgical approach, which involved placing a tissue plug at the inferior aspect of the fourth ventricle (the obex) to prevent CSF transmission. However, subsequent studies suggested that the success of such procedures may have been more attributable to posterior fossa decompression and intradural exposure, rather than the plug itself. Postmortem examinations have demonstrated that in most cases of Chiari-related syringomyelia, there is no anatomical connection between the fourth ventricle and the syrinx, a finding corroborated by modern MRI studies [10,11].

### 4.2. Primary Spinal Syringomyelia (PSS)

Primary spinal syringomyelia refers to cases where the syrinx develops due to pathology intrinsic to the spine, without associated abnormalities at the craniocervical junction. This form of syringomyelia is considerably less common than Chiari-related variants.

Recognized causes of PSS include the following:

1. Spinal trauma.
2. Arachnoid cysts.
3. Post-infectious scarring from meningitis or subarachnoid hemorrhage [11].

While intraspinal neoplasms may present with associated cystic cavities, they are not typically classified as true syringomyelia unless they significantly compromise CSF flow by narrowing the subarachnoid space. Additionally, structural anomalies such as spondylotic ridges or intervertebral disc herniations may create partial CSF flow obstruction, contributing to syrinx formation through pressure differentials [4]. In rare cases, syrinx rupture and subsequent communication with the subarachnoid space can occur, especially following spinal cord injury [12].

### 4.3. Tethered Cord Syndrome and Spina Bifida

An important, though sometimes under-recognized, subset of syringomyelia cases is associated with congenital spinal dysraphisms, particularly tethered cord syndrome (TCS). TCS may arise as part of spina bifida occulta or aperta, conditions characterized by abnormal fixation of the spinal cord resulting in restricted movement. This abnormal tension can disrupt cerebrospinal fluid dynamics, contributing to the formation of a syrinx and the development of neuromuscular scoliosis. It is crucial to recognize that, in patients presenting with neuromuscular scoliosis and syringomyelia, especially in the context of underlying spinal dysraphism, comprehensive evaluation for TCS and spina bifida should be undertaken. In such cases, neurosurgical intervention aimed at untethering the spinal cord is recommended as the initial step. Only after the underlying tethering has been addressed should orthopedic correction of the spinal deformity be considered, as this sequence reduces the risk of neurological deterioration and improves surgical outcomes [13,14].

### 4.4. Risk Factors for Syringomyelia

Beyond well-established etiologies, several risk factors have been identified that may increase susceptibility to syringomyelia. Among iatrogenic factors, conditions such as increased surgical site bleeding or traumatic lumbar punctures may promote arachnoid fibrosis, which can impair CSF circulation. Post-traumatic syringomyelia is of particular clinical interest. The most significant risk factor appears to be complete spinal cord injury, defined as grade A on the American Spinal Injury Association (ASIA) Impairment Scale, which is associated with a twofold increase in the risk of syrinx development. Other recognized contributors include the following:

1. Post-traumatic kyphosis exceeding 15°.
2. Spinal canal stenosis greater than 25%.

Importantly, the presence of these risk factors does not necessarily mandate surgical intervention but should prompt careful monitoring and radiological evaluation [15].

## 5. Pathophysiology

### 5.1. Theoretical Perspectives on Syringomyelia Formation

Multiple theories have been proposed to explain syringomyelia pathophysiology, but no single model accounts for all cases. Contemporary views focus on altered cerebrospinal fluid (CSF) dynamics and pressure gradients within the spinal cord, particularly due to obstruction at key anatomical sites. Classic theories—including those by Gardner, Williams, Heiss, and Oldfield—highlight the role of disrupted CSF flow at the foramen magnum and the impact of cerebellar tonsillar herniation. Although early hypotheses varied, it is now widely accepted that partial CSF obstruction leads to pressure differentials, promoting syrinx formation and progression [16,17,18].

### 5.2. Mechanisms of Syrinx Formation

Syrinx formation is primarily attributed to altered CSF flow dynamics, often due to mechanical obstruction from Chiari malformations, spinal trauma, tumors, or arachnoid adhesions. These obstructions create pressure gradients that drive CSF into the spinal cord, resulting in cavity formation and progressive tissue damage. In Chiari-related syringomyelia, the exact mechanisms remain incompletely understood, but several hypotheses have been proposed:

1. Water Hammer Theory suggests that partial obstruction at the fourth ventricle redirects systolic CSF pulses down a patent central canal, leading to syrinx formation [19].
2. Cranial–Spinal Pressure Dissociation Theory proposes that a caudal CSF block results in higher intracranial pressure relative to spinal intrathecal pressure, forcing CSF downward into the central canal, thereby producing communicating syringomyelia. However, this proposed communication is rarely seen in MRI or autopsy studies.
3. Ball and Dayan Theory posits that intermittent increases in spinal CSF pressure, caused by cerebellar tonsil obstruction during activities like coughing or Valsalva maneuver, force CSF through extracellular spaces along the spinal cord surface, initiating syrinx formation [20].
4. Piston Theory suggests that cerebellar tonsils act as a piston, creating pressure waves in the spinal subarachnoid space that drive CSF into the perivascular or subpial spaces, ultimately contributing to syrinx expansion [21].
5. Perivascular Flow Disruption Theory proposes that CSF normally flows along perivascular spaces, but disruption of this flow, as seen in Chiari malformations, can lead to increased inflow or reduced outflow, resulting in syrinx development and enlargement [22].

Spontaneous syrinx resolution, although rare, is hypothesized to occur when tonsillar descent decreases slightly or CSF flow improves at the craniocervical junction, restoring pressure equilibrium. This supports Stoodley’s model, in which syrinx volume reflects the balance between CSF inflow and outflow [23].

Syringomyelia can also develop in individuals without known risk factors or in association with spinal cord tumors, trauma, or scarring. Up to 30% of spinal cord tumors are associated with syrinx formation, likely due to CSF flow disruption. The gray matter, located centrally in the spinal cord, is often involved early, as it contains the neuronal cell bodies, while the white matter, composed of axons, is affected as the syrinx expands. The central canal, which houses the CSF, is located at the core of the gray matter [15].

### 5.3. Impact on the Spinal Cord

The expanding syrinx can cause progressive neurological damage by compressing spinal cord structures, particularly in the cervical and thoracic regions, which are responsible for motor and sensory innervation of the upper limbs. This pressure can disrupt local neural circuits, leading to autonomic dysfunction, sensory loss, and muscular weakness [24]. The clinical impact is highly dependent on the size, location, and progression rate of the syrinx, as well as the extent of spinal cord involvement.

## 6. Orthopedic Manifestations and Musculoskeletal Involvement

Orthopedic manifestations of syringomyelia are often under-recognized but may provide critical early diagnostic clues. Common findings include scoliosis, pes cavus, progressive gait disturbances, and neurogenic arthropathies (Charcot joints), especially of the shoulder and elbow [25]. These complications typically result from disruption of motor and sensory spinal pathways and may represent the first clinical presentation in some patients. Early orthopedic imaging plays a pivotal role in the assessment of patients presenting with atypical musculoskeletal symptoms—such as unexplained joint destruction, rapidly progressive scoliosis, or neurogenic arthropathy. Prompt radiographic and advanced imaging studies, including MRI, can reveal underlying spinal cord pathology such as syringomyelia at an early stage, enabling multidisciplinary intervention and reducing the risk of delayed diagnosis and irreversible complications [24,25,26,27].

### 6.1. Scoliosis in Syringomyelia

Among the orthopedic complications, scoliosis holds particular significance. Multiple studies have demonstrated a strong association between syringomyelia and scoliosis, particularly in cases involving Chiari malformation. Scoliosis has been reported in 25% to 74% of patients with syringomyelia, while syringomyelia is found in up to 9.7% of patients with scoliosis undergoing preoperative imaging [26,27].

While often considered a consequence of asymmetrical paraspinal muscle innervation caused by syrinx-induced motor neuron dysfunction, some authors have also proposed an etiological link to chronic atlantoaxial instability, where syringomyelia and scoliosis may represent adaptive spinal responses to subtle instability at the craniocervical junction [28].

The characteristics and extent of the syrinx cavity appear to correlate with neurological involvement and scoliosis progression. A syrinx width greater than 4 mm is commonly regarded as a threshold for neurosurgical evaluation, as larger cavities are more likely to cause progressive neurological symptoms and spinal deformity [26]. The configuration of the syrinx and its relationship to curve convexity may influence progression patterns, although not uniformly [26,27].

Management strategies must be individualized. In patients with significant neurological symptoms or large syrinx cavities, posterior fossa decompression or syrinx shunting is typically considered prior to scoliosis correction [26,27]. In neurologically stable patients with minimal syrinx diameter, orthopedic correction with neuromonitoring may proceed safely without prior neurosurgical intervention. Several studies report favorable outcomes in scoliosis correction following neurosurgical stabilization, with low rates of progression and neurological deterioration when appropriate multidisciplinary planning is undertaken [26,27].

Importantly, MRI should be strongly considered in scoliosis patients presenting with atypical curve patterns, rapid progression, neurological symptoms, or significant back pain out of proportion to deformity. In such cases, a high index of suspicion for underlying intraspinal pathology—including syringomyelia—is warranted.

### 6.2. Neurological Features with Musculoskeletal Implications

**Figure 2:**
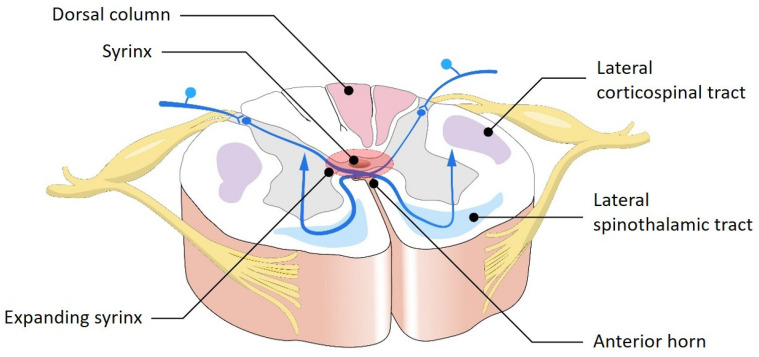
Cross-sectional schematic of the spinal cord illustrating the anatomical impact of syringomyelia. The expanding syrinx centrally disrupts the decussating fibers of the lateral spinothalamic tracts, leading to dissociated sensory loss—typically affecting pain and temperature sensation. The dorsal columns, lateral corticospinal tracts, and anterior horns may also become progressively compromised as the syrinx enlarges, resulting in motor weakness and additional sensory deficits.

As a syrinx expands, it can disrupt the decussating fibers of the lateral spinothalamic tracts, which are responsible for transmitting pain and temperature sensation (Figure 2).

**Figure 3:**
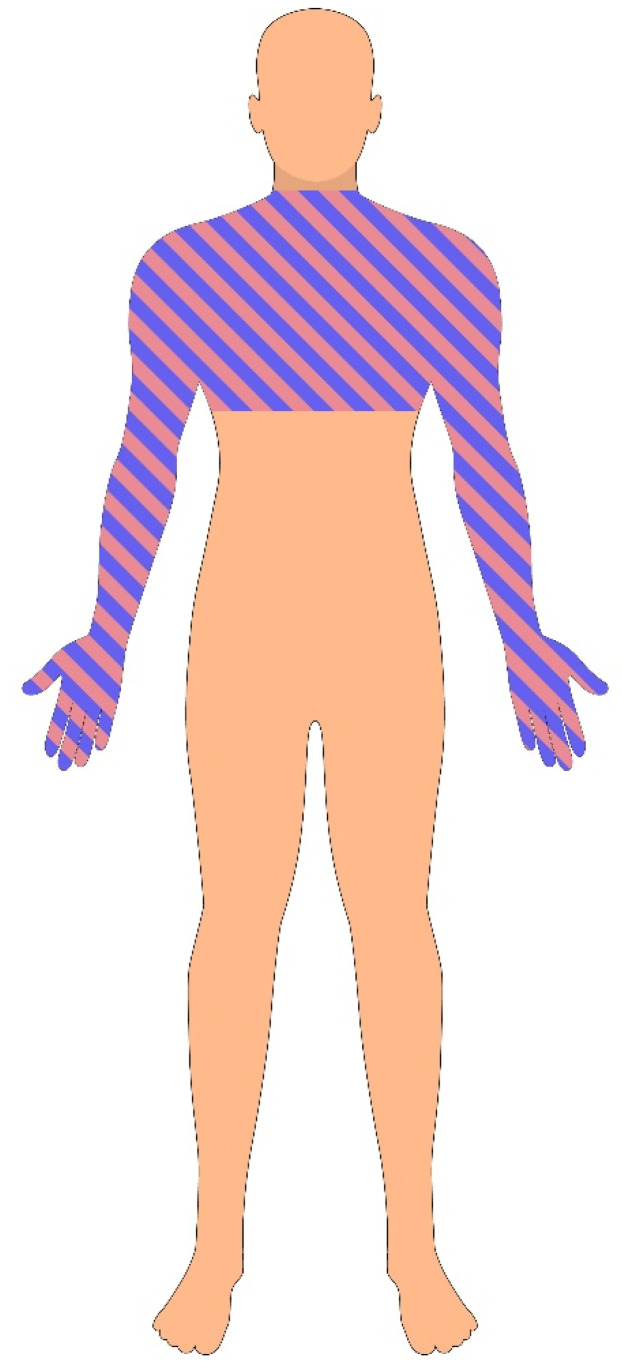
Cross-sectional spinal cord diagram demonstrating the anatomical disruption caused by an expanding syrinx. The syrinx originates centrally and enlarges outward, compressing surrounding neural structures. Early involvement of the decussating spinothalamic fibers explains the characteristic dissociated sensory loss (pain and temperature), while further expansion may affect the lateral corticospinal tracts (motor pathways) and anterior horn cells (lower motor neurons), contributing to progressive motor deficits.

Clinical symptoms are often diverse and may mimic other neurological conditions, ranging from atypical chest pain to multiple sclerosis. The pattern of sensory complaints typically follows a dermatomal distribution, often in the cervical or thoracic regions. Early manifestations may include unilateral hypesthesia in the arm, hand, or axilla, accompanied by reduced reflexes. A hallmark sensory presentation is the “cape-like” distribution of sensory loss, involving the nape of the neck, shoulders, and upper arms (Figure 3).

If the syrinx extends anteriorly, it may produce lower motor neuron weakness, while lateral expansion can result in upper motor neuron signs below the level of the lesion. As the cavity continues to enlarge, patients may develop bilateral paresthesia and progressive weakness. Symptoms may initially appear subtle—such as an inability to sense heat from a coffee mug—before evolving into more disabling features like difficulty climbing stairs due to spasticity and motor weakness.

In cases where the syrinx extends into the brainstem, symptoms may involve cranial nerve dysfunction, producing ipsilateral facial sensory loss, dysphagia, tongue weakness, ptosis, miosis, or diplopia. Autonomic involvement may manifest as loss of facial sweating or altered temperature sensation. Additionally, gastrointestinal symptoms such as nausea, vomiting, feeding difficulties, weight loss, or visceral contractions may arise due to the involvement of esophageal and autonomic visceral reflex centers.

### 6.3. Charcot Joints and Neurogenic Arthropathy

Syringomyelia may also present with orthopedic complications due to its impact on both motor and sensory pathways. One of the most distinctive manifestations is neurogenic arthropathy (Charcot joint), most commonly affecting the shoulder, although other joints such as the elbow may also be involved [25]. This progressive condition is characterized by soft tissue swelling, bone resorption, joint space narrowing, intra-articular calcification, and eventual joint subluxation or dislocation. Syringomyelia is considered the second most common cause of Charcot joint, although its exact pathological mechanism remains unclear.

In many cases, patients are first referred to an orthopedic surgeon, often before a neurological diagnosis has been established. Despite its under-recognition, syringomyelia is not as rare as it may be perceived. However, its clinical presentation can be highly variable, and diagnosis may be overlooked if evaluation focuses solely on classic deformities such as scoliosis or pes cavus. Localized pain in the head, neck, trunk, or limbs—especially when exacerbated by exertion—should raise clinical suspicion.

Certain clinical clues may aid diagnosis. A history of birth trauma, especially when accompanied by later-developing spasticity, may be relevant. Additional findings include nystagmus, dissociated sensory loss, muscle atrophy, and lower limb spasticity, along with the presence of Charcot joints. Radiographic findings may also support the diagnosis—such as spinal canal enlargement and degenerative changes in the cervical vertebrae. A pathological expansion of the spinal canal is considered significant when, at the C5 level, its diameter exceeds the vertebral body by 6 mm or more [29].

Interestingly, syringomyelia symptoms may be triggered or exacerbated by acceleration–deceleration forces such as roller coaster rides, intense coughing, minor trauma, or even sneezing. These events may transiently elevate CSF pressure and provoke syrinx expansion, resulting in acute symptom onset or progression. It is hypothesized that such CSF dynamics can facilitate fluid movement into the cavity, worsening spinal cord compression and promoting neurological deterioration.

### 6.4. Illustrative Clinical Case

A notable case from our clinical experience illustrates the orthopedic implications of advanced syringomyelia. A 40-year-old woman, morbidly obese and a mother of three, had experienced progressive gait disturbance over several years. Neurological evaluation revealed a type IV idiopathic syringomyelia involving the entire thoracic spinal cord, with extension into the proximal cervical segments. Due to ongoing neurological decline and emerging urinary dysfunction, she underwent subarachnoid–peritoneal shunt surgery at the D11–D12 level three years ago. Unfortunately, postoperative outcomes were unfavorable, and her condition progressed to complete paraplegia with loss of bowel and bladder control. Although additional syrinx decompression surgery was proposed, the patient declined further intervention. Over time, her neurological status continued to deteriorate, and she recently developed progressive weakness in the left upper limb, further complicating her clinical course.

**Figure 4:**
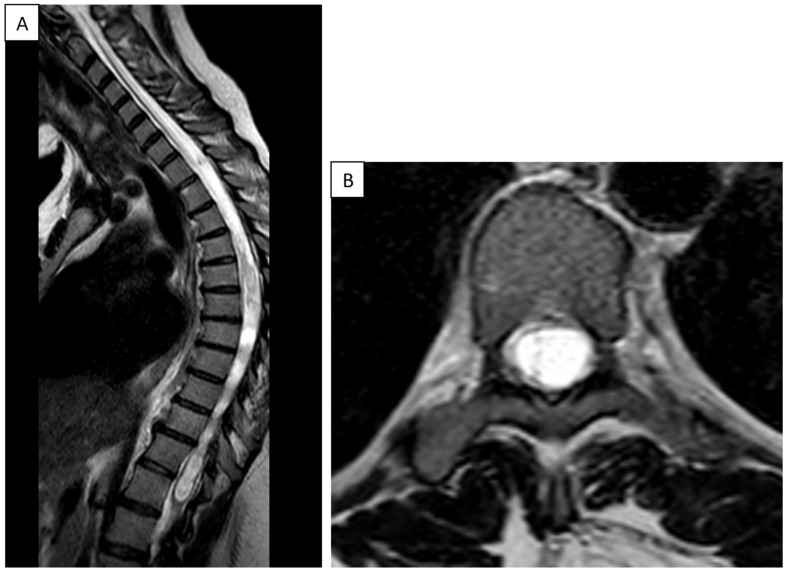
MRI of the thoracic spine in a patient with syringomyelia. (**A**) Sagittal T2-weighted image demonstrating an elongated, hyperintense intramedullary cavity consistent with a syrinx extending along the thoracic spinal cord. (**B**) Axial T2-weighted image showing a well-defined syrinx centrally located within the spinal cord, causing expansion of the cord parenchyma.

She was subsequently admitted to our department with a six-week history of progressive pain and functional impairment of the left shoulder, with no history of trauma. On examination, the shoulder appeared swollen and mildly warm, with complete loss of active movement and significant limitation of passive mobility, especially in abduction and external rotation. Laboratory studies revealed a normal white blood cell count and a mildly elevated C-reactive protein (CRP) level of 9 mg/L. MRI demonstrated significant thoracic and cervical spine syringomyelia (Figure 4).

**Figure 5:**
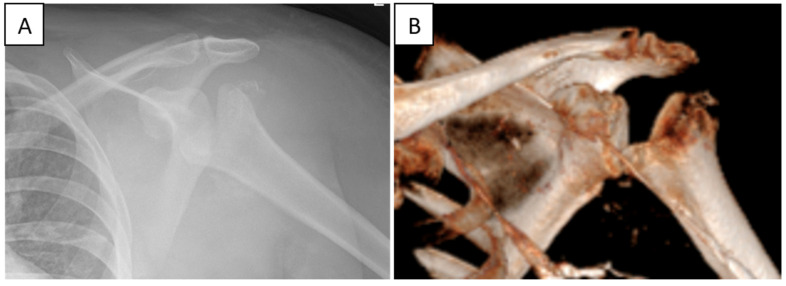
Radiographic and 3D imaging of a neurogenic (Charcot) shoulder joint in a patient with syringomyelia. (**A**) Plain radiograph showing marked destruction of the humeral head, joint subluxation, and loss of normal articular contours. (**B**) Three-dimensional reconstructed CT image highlighting severe bony fragmentation, deformity, and intra-articular debris consistent with advanced neuropathic arthropathy.

Radiographs (Figure 5A) and a CT scan of her left shoulder (Figure 5B), showed severe humeral head destruction.

Joint aspiration yielded reactive synovial fluid without evidence of infection; Gram stain and cultures were negative.

The clinical and radiological findings were consistent with a Charcot joint of the shoulder, a rare yet well-recognized complication of syringomyelia involving the cervical and upper thoracic spinal cord. This case underscores the importance of orthopedic vigilance in neurologically impaired patients, in whom joint destruction can progress silently due to loss of proprioceptive and nociceptive feedback.

## 7. Diagnosis

Diagnosis of syringomyelia can be challenging due to its diverse clinical presentations and often insidious onset. While it is frequently discovered incidentally during imaging for unrelated complaints, a structured diagnostic approach remains essential. The following subheadings outline key aspects of clinical assessment and imaging modalities used in the diagnosis of this condition.

### 7.1. Clinical Presentation

Although syringomyelia is often diagnosed incidentally, most symptomatic patients initially present with sensory disturbances, most commonly pain and impaired temperature sensation. The widespread use of magnetic resonance imaging (MRI) for the evaluation of back and neck pain has contributed to the increasing detection of this condition. The natural history of syringomyelia is highly variable, typically characterized by periods of clinical stability interspersed with progression. In most cases, the clinical course evolves gradually over months to years, often beginning with a phase of rapid decline, followed by a slower, more protracted deterioration [30].

Post-traumatic syringomyelia, in particular, has been the subject of extensive research. Patients with previously stable spinal cord injuries may develop delayed neurological deterioration, with clinical symptoms typically manifesting approximately nine years after the initial trauma [31]. Common symptoms include motor weakness, chronic pain, and altered sensation. However, syringomyelia lacks a pathognomonic symptom complex and may mimic a variety of neurological syndromes, complicating early diagnosis.

Among recognized clinical syndromes, central cord syndrome is most commonly associated with syringomyelia. This syndrome arises when the syrinx primarily affects the central gray matter of the spinal cord. Clinical features often include temperature dysregulation, ulcer formation, and signs of lower motor neuron dysfunction, such as muscle atrophy, flaccid weakness, and diminished reflexes at the level of the lesion.

A study by Bogdanov et al. explored whether Chiari-related and non-Chiari syringomyelia present with differing symptom profiles. The findings revealed similar symptom frequencies in both groups, with segmental sensory loss (93%), muscle atrophy (60%), and pyramidal signs (82%) being the most prevalent findings [18].

- Segmental sensory loss refers to localized sensory deficits corresponding to the dermatomal level of spinal cord involvement.
- Pyramidal signs indicate upper motor neuron dysfunction, often presenting as spasticity, hyperreflexia, and a positive Babinski sign, reflecting disruption of the corticospinal tract.

Not all patients present with the classic triad of burning pain, segmental weakness, and dissociated sensory loss.

- Dissociated sensory loss is a hallmark of syringomyelia and describes the selective loss of pain and temperature sensation, with preservation of light touch, vibration, and proprioception. This pattern occurs because spinothalamic fibers, which carry pain and temperature sensations, cross within the central spinal cord and are affected early by syrinx expansion, whereas dorsal column pathways—carrying vibration and proprioception signals—are located more peripherally and remain intact.

Early symptoms often play a critical role in predicting long-term outcomes, underscoring the importance of timely diagnosis and intervention. In centrally located syrinxes, involvement of the gray commissures in lamina X contributes directly to dissociated sensory loss. Additional manifestations may include autonomic bladder dysfunction, typically resulting from disruption of descending autonomic pathways. Bowel dysfunction, however, tends to emerge in later stages due to the involvement of laminae VII (Clarke’s column)—which contains preganglionic autonomic neurons—and laminae VIII and IX, which are associated with skeletal motor control.

Other frequently encountered symptoms include the following:

- Radicular pain: Pain radiating along a dermatome due to nerve root irritation.
- Gait ataxia: Impaired coordination and unsteady walking due to sensory or motor deficits.
- Dysesthesias: Abnormal sensations often described as burning, tingling, or electric-shock-like pain.
- Spasms and spasticity: Involuntary muscle contractions or stiffness due to upper motor neuron involvement.
- Autonomic dysreflexia: A potentially life-threatening condition characterized by sudden hypertension, bradycardia, and profuse sweating, often triggered by noxious stimuli below the level of spinal cord injury.
- Neuropathic pain: Chronic pain caused by direct injury to the nervous system, often disproportionate to physical findings [6].

Spasticity often progresses with the advancement of syringomyelia and may significantly impact functional mobility. Nonetheless, the disease typically progresses slowly and insidiously, and many patients exhibit relatively stable neurological signs for extended periods [32].

### 7.2. Imaging Studies

While clinical suspicion remains the cornerstone of diagnosis, imaging plays a pivotal role in confirming syringomyelia and assessing its underlying cause. Syringomyelia should be considered in patients with the following:

1. Persistent spinal cord lesions;
2. A history of spinal surgery or trauma;
3. Gradually progressive neurological symptoms;
4. Abrupt symptom exacerbation without a clear alternative diagnosis.

Although clinical and imaging findings can support diagnosis, there remains no universally accepted gold standard for confirming syringomyelia [16].

### 7.3. MRI and Cine MRI

Magnetic resonance imaging (MRI) is now the preferred imaging modality for diagnosing syringomyelia. MRI provides detailed visualization of the spinal cord, syrinx cavity, and associated anomalies such as Chiari malformations. In selected cases, cine MRI—a dynamic phase-contrast technique used to assess pulsatile cerebrospinal fluid (CSF) flow—may aid in evaluating CSF dynamics. However, its routine use in syringomyelia assessment remains limited. Some studies, including those by Inoue et al., have attempted to correlate syrinx morphology (size, shape, and location) with the site of obstruction, but the ability of MRI to localize the exact point of CSF flow impairment remains limited [21]. Although cine MRI may offer better functional imaging than CT-myelography, it is still not consistently reliable in pinpointing flow blockages [18]. Furthermore, there is currently no standardized data on the sensitivity and specificity of these techniques in syringomyelia diagnosis.

### 7.4. Diagnostic Workup: Stepwise Flowchart

**Figure 6:**
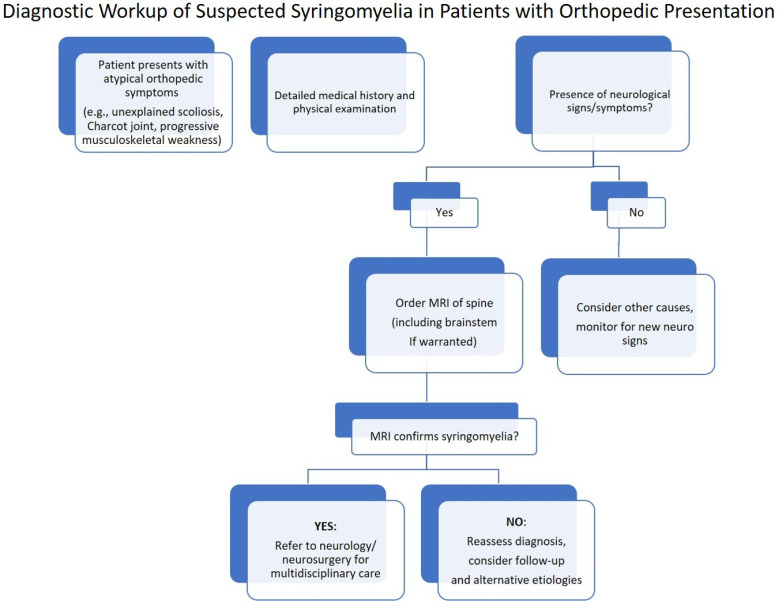
Suggested flowchart for the diagnostic workup of suspected syringomyelia in patients presenting with orthopedic symptoms. Multidisciplinary collaboration is recommended at each step to optimize patient outcomes.

To enhance clinical clarity, we have included a flowchart outlining the recommended diagnostic pathway for suspected syringomyelia in patients presenting with orthopedic manifestations. This stepwise approach highlights key clinical decision points and supports effective multidisciplinary management (see Figure 6).

## 8. Management

The management of syringomyelia typically follows one of two main approaches: conservative treatment or surgical intervention [32,33]. This depends on the severity of symptoms, radiological findings, and progression of neurological deficits. While surgery is often indicated in cases of significant spinal cord compression or rapidly evolving neurological impairment, conservative treatment remains the first-line approach for patients with mild or stable disease, with a focus on symptom control and quality of life enhancement.

### 8.1. Conservative Management

Conservative treatment includes medical management for symptom relief and physical therapy to support function and mobility.

1. Medical Management

Pharmacologic therapy targets neuropathic pain, spasticity, and associated neurological symptoms, and involves several classes of medications:

A. Anticonvulsant Agents: Gabapentin and pregabalin have been shown to improve neuropathic pain, including hyperalgesia and allodynia, through modulation of voltage-gated calcium channels [34,35,36,37,38]. Pregabalin has also demonstrated efficacy in reducing phantom scratching and other sensory disturbances [35].
B. Proton Pump Inhibitors (e.g., Omeprazole): Anecdotal reports have suggested that PPIs may reduce CSF production, potentially relieving syringomyelia symptoms, though evidence is limited and clinical studies have failed to confirm a significant CSF-lowering effect [39,40,41].
C. Nonsteroidal Anti-Inflammatory Drugs (NSAIDs): Some studies suggest a role for COX-2 inhibitors (e.g., carprofen, meloxicam, deracoxib, and firocoxib) in the relief of neuropathic pain in syringomyelia, though the results remain debated [34,40].
D. Carbonic Anhydrase Inhibitors (e.g., Acetazolamide): These agents may reduce CSF flow and have been proposed as adjuncts in conservative management [41].
E. Corticosteroids: Prednisolone and methylprednisolone may help reduce both pain and neurologic symptoms, possibly by modulating substance P and inflammatory mediators. However, long-term use is limited by side effects such as immunosuppression, weight gain, and metabolic disturbances [17,42].
F. Opioids: Opioid medications are occasionally used, particularly in the postoperative setting. Methadone, with its NMDA receptor antagonist activity, may provide better control of intractable neuropathic pain, although tolerance and dependency issues limit their long-term use [43,44,45].
G. Diuretics (e.g., Furosemide): Furosemide may help lower intracranial pressure, although some studies suggest its effects may be secondary to diuresis rather than direct CSF pressure reduction, and results remain inconclusive [17,45,46].
2. Management of Spasticity

Spasticity, often a consequence of upper motor neuron involvement, requires individualized assessment and treatment. Symptoms may include spasms, clonus, hyperreflexia, and muscle co-contraction [47,48]. This includes the following:

A. Baclofen (a GABA receptor agonist)—first-line treatment for spasticity [49,50].
B. Tizanidine, clonidine, dantrolene, and benzodiazepines—adjunctive agents with variable efficacy and tolerability [48,49,50].
C. Botulinum toxin injections—effective in focal spasticity, often used in combination with physiotherapy [49].
D. Intrathecal baclofen, delivered via an implantable pump, may be considered in generalized, drug-refractory spasticity [48,49].
3. Physical Therapy and Rehabilitation

Physical therapy is a core component of conservative care. Interventions may include the following:

- Stretching and strengthening exercises.
- Weight-bearing and postural training.
- Orthotic support (e.g., ankle–foot orthoses).
- Serial casting.
- Cryotherapy, thermotherapy, and electrical stimulation.

These techniques can help modulate spasticity, improve functional mobility, and maintain independence [51].

Although robust data are limited, postoperative physical rehabilitation and conservative physiotherapy in non-surgical candidates have shown beneficial effects on functional outcomes and quality of life [52].

### 8.2. Surgical Management

Surgical intervention remains the mainstay of treatment in patients with syringomyelia presenting with progressive neurological symptoms or functional impairment, particularly when a syrinx cavity is associated with mechanical obstruction. While some researchers advocate conservative monitoring in stable cases due to the often slow progression of motor deficits [53], others support early surgical intervention to prevent irreversible neurological deterioration [54].

The primary goal of surgical treatment is to address the underlying cause of syrinx formation, restore cerebrospinal fluid (CSF) flow, and alleviate spinal cord compression [55]. The selection of surgical technique depends on the etiology of syringomyelia, anatomical location, and individual patient characteristics.

A. Suboccipital Decompression (Posterior Fossa Decompression): In patients with Chiari malformation type I (CM-I), the most commonly performed procedure is posterior fossa decompression (PFD) or craniocervical decompression, which aims to reestablish normal CSF circulation by removing bone at the foramen magnum and often includes dural opening and duraplasty [56,57,58,59]. There is ongoing debate regarding the extent of decompression, particularly the need to open the dura. Some surgeons advocate dural opening with a patch graft, citing its importance in identifying and releasing arachnoid scarring or other obstructions, which are present in up to 55% of cases [57]. Others argue that dural opening may increase the risk of complications. Intraoperative ultrasonography has been proposed to optimize decompression and tailor surgery to individual CSF flow dynamics [17].
B. Shunt Placement: In cases where decompression does not result in syrinx regression, or in post-traumatic syringomyelia, shunt placement may be considered. Various shunt types include the following:
  - Syringosubarachnoid shunt.
  - Syringoperitoneal shunt.
  - Syringopleural shunt.

These techniques divert CSF from the syrinx into extradural or extracorporeal compartments, alleviating pressure and preventing further expansion [45,59]. Syringosubarachnoid shunting, particularly when performed using minimally invasive surgical (MIS) techniques, has been associated with reduced postoperative complications, such as CSF leaks, wound infections, and prolonged recovery [45,59]. However, shunting is not without limitations. Shunt obstruction, infection, or failure requiring revision surgery is common, and direct syrinx drainage is often associated with high failure rates and potential for cord tethering [45,59].

C. Spinal Cord Surgery and Direct Decompression: In select cases, direct surgical decompression of spinal cord cavities may be required. Techniques include restoration of subarachnoid pathways or syrinx drainage via myelotomy. However, these procedures pose significant risks, including dorsal column injury, especially in patients who retain some neurological function [60]. Moreover, septated syrinx cavities may limit the effectiveness of shunting, and unless the underlying cause of fluid accumulation is addressed, new cavities may continue to form despite successful decompression [60].
D. Etiology-Directed Surgery: In cases where the syrinx is caused by an identifiable compressive lesion, such as a tumor or scar tissue, removal of the obstruction can restore CSF flow and often lead to syrinx resolution [61]. Tumor resection, when feasible, remains the primary approach, although radiation therapy may be considered in certain scenarios.
E. Drainage Procedures: In patients with idiopathic or progressively enlarging syrinx cavities, drainage via stent or shunt placement may be considered. A stent allows for internal diversion of syrinx fluid, while a shunt system, typically composed of a catheter and valve mechanism, diverts fluid to pleural or peritoneal spaces [62]. These procedures may stabilize or improve symptoms, although long-term success varies, and some patients may experience recurrence requiring reoperation [63].

Following surgery, MRI is used to monitor the syrinx cavity, with reduction or stabilization considered indicative of a favorable response. Although surgery can significantly improve symptoms and slow disease progression, recurrence remains possible, underscoring the need for ongoing clinical and radiological follow-up.

## 9. Treatment Considerations in Charcot Shoulder

The clinical approach to neuropathic shoulder arthropathy—particularly in the context of syringomyelia—requires a high index of suspicion, comprehensive neurological assessment, and appropriate imaging to exclude other etiologies such as infection, neoplasm, or inflammatory arthropathy. As highlighted in the literature [64,65], the orthopedic surgeon is often the first to evaluate such patients due to joint-related complaints, despite the underlying neurologic pathology being the primary cause.

Conservative management remains the cornerstone of initial treatment and includes patient education, joint protection, weight-bearing restriction, immobilization, and physical therapy focused on preserving passive range of motion and limiting further joint destruction. Nonsteroidal anti-inflammatory drugs (NSAIDs) may be used to address secondary synovitis. Once the active neurologic process is stabilized—often following neurosurgical decompression—surgical options may be considered for select patients. These include joint-preserving procedures such as shoulder resurfacing arthroplasty or humeral head replacement, particularly when the joint remains unstable or severely dysfunctional despite conservative care. However, surgical intervention should be approached with caution, considering the inherent risks of instability, infection, and prosthetic failure due to the loss of protective proprioception and muscle control.

### Back to Our Patient

The patient’s neurological status was defined as a failed syrinx decompression, with persistent and irreversible loss of neurological function. Additionally, the patient declined further spinal surgical intervention. The resulting chronic paraplegic state, accompanied by recent onset of left hand weakness, significantly influenced clinical decision-making. Given the extent of neurological damage and the advanced stage of joint destruction, it was concluded that orthopedic surgery would offer limited benefit. The presence of a neuropathic joint, combined with complete paralysis of the surrounding musculature, predicted a high risk of surgical failure. As such, a conservative management strategy was adopted, focusing on pain control, joint protection, and preservation of functional independence. This case underscores the importance of individualized treatment planning, particularly when orthopedic complications coexist with severe and irreversible neurological impairment.

## 10. Summary and Conclusions

Syringomyelia remains a complex and multifaceted neurological disorder characterized by the development of a fluid-filled cavity within the spinal cord. Its clinical presentation is often subtle and variable, ranging from mild sensory disturbances to severe motor deficits and orthopedic complications, such as Charcot arthropathy. The diagnostic process can be challenging, as symptoms frequently mimic other neurological conditions and may initially present to non-neurological specialists, particularly in orthopedic settings.

The orthopedic manifestations of syringomyelia are often under-recognized, yet they may be among the earliest signs of the disease. Neurogenic joint arthropathy, postural deformities, pes cavus, scoliosis, and progressive gait disturbances can be key clinical clues, often prompting referral to orthopedic care. A high index of suspicion is essential, as failure to identify the underlying neurological etiology may delay appropriate treatment and result in irreversible damage.

One particularly rare but illustrative manifestation is neuropathic shoulder arthropathy, which can result in silent and progressive joint destruction due to impaired protective sensation and proprioception. Such cases may initially present with pain, stiffness, or loss of shoulder function without a clear traumatic history, underscoring the need for orthopedic teams to consider syringomyelia in the differential diagnosis. Early recognition of this phenomenon is critical to avoid misdiagnosis and to guide appropriate multidisciplinary management.

The pathophysiology of syringomyelia is rooted in abnormal cerebrospinal fluid dynamics, often secondary to structural abnormalities such as Chiari malformation, spinal trauma, tumors, or arachnoid adhesions. A growing understanding of these mechanisms has advanced both conservative and surgical management strategies.

Management approaches must be individualized. While conservative treatment, including pharmacologic symptom control and physical rehabilitation, remains the first-line strategy in patients with stable or mild symptoms, surgical intervention becomes imperative in progressive cases or those with identifiable structural compression. Posterior fossa decompression, shunting procedures, and minimally invasive spinal techniques each have a defined role depending on the underlying cause and clinical course.

Ultimately, timely recognition, multidisciplinary evaluation, and individualized treatment planning are essential to optimizing outcomes in patients with syringomyelia. Incorporating orthopedic perspectives early in the diagnostic and therapeutic process is particularly important, especially when musculoskeletal complaints are the first clinical manifestation. Long-term follow-up is critical, as syringomyelia may progress or recur—even after apparently successful treatment, as demonstrated in our case. Continued research is needed to refine treatment algorithms and enhance diagnostic accuracy, particularly in early-stage or asymptomatic patients. Recognizing and appropriately managing underlying conditions such as tethered cord syndrome and spina bifida is essential in the comprehensive care of patients with syringomyelia and neuromuscular scoliosis. Early diagnosis and timely neurosurgical intervention can optimize neurological outcomes and ensure safer orthopedic correction, when indicated.

## 11. Key Messages

- Syringomyelia is a complex spinal cord disorder characterized by fluid-filled cavities within the spinal cord, often resulting from disrupted cerebrospinal fluid (CSF) dynamics due to Chiari malformation, trauma, or intradural lesions.
- Orthopedic manifestations are frequent but under-recognized and may represent the earliest clinical signs. These include neurogenic arthropathy (Charcot joints)—most notably involving the shoulder—as well as pes cavus, scoliosis, gait disturbances, and unexplained limb weakness.
- Neuropathic shoulder arthropathy, though rare, can lead to progressive joint destruction due to impaired proprioception and pain perception. Early identification is crucial to prevent irreversible structural damage.
- Clinical symptoms of syringomyelia vary widely, often mimicking other neurological or orthopedic conditions. Early signs may include dissociated sensory loss, spasticity, paresthesia, or segmental motor weakness.
- Diagnosis requires a high index of suspicion, especially in orthopedic settings, where patients may present with joint pain or dysfunction without a history of trauma or infection.
- Conservative treatment—including analgesics, anticonvulsants, physical therapy, and joint protection—remains the first-line strategy for both neurologic and orthopedic manifestations.
- Surgical intervention, including posterior fossa decompression, syrinx shunting, or lesion removal, is indicated in progressive cases and should be tailored to the underlying etiology.
- Long-term follow-up is essential, not only to assess neurological stability and syrinx progression, but also to monitor orthopedic complications, especially in joints affected by neurogenic arthropathy.
